# Environmental Health Literacy as Knowing, Feeling, and Believing: Analyzing Linkages between Race, Ethnicity, and Socioeconomic Status and Willingness to Engage in Protective Behaviors against Health Threats

**DOI:** 10.3390/ijerph19052701

**Published:** 2022-02-25

**Authors:** Andrew R. Binder, Katlyn May, John Murphy, Anna Gross, Elise Carlsten

**Affiliations:** 1 Center for Human Health & the Environment, North Carolina State University, Raleigh, NC 27695, USA; kmay2@ncsu.edu (K.M.); jmurphy5@ncsu.edu (J.M.); emcarlst@ncsu.edu (E.C.); 2 Department of Communication, North Carolina State University, Raleigh, NC 27695, USA; 3 Center for Health and Equity Research, University of North Carolina at Chapel Hill, Chapel Hill, NC 27599, USA; anna_gross@med.unc.edu

**Keywords:** environmental health literacy, health disparities, COVID-19, PFAS, race and ethnicity, socioeconomic status, efficacy, knowledge sufficiency, factual knowledge

## Abstract

This study investigates the relationships between environmental health literacy, the characteristics of people (race, ethnicity, and socioeconomic status) associated with health disparities, and people’s willingness to engage in protective behaviors against environmental health threats. Environmental health literacy is a framework for capturing the continuum between the knowledge of environmental impacts on public health, and the skills and decisions needed to take health-protective actions. We pay particular attention to three dimensions of environmental health literacy: factual knowledge (knowing the facts), knowledge sufficiency (feeling ready to decide what to do), and response efficacy (believing that protective behaviors work). In June 2020, we collected survey data from North Carolina residents on two topics: the viral infection COVID-19 and industrial contaminants called per- and polyfluoroalkyl substances (PFAS). We used their responses to test stepwise regression models with willingness to engage in protective behaviors as a dependent variable and other characteristics as independent variables, including environmental health literacy. For both topics, our results indicated that no disparities emerged according to socioeconomic factors (level of education, household income, or renting one’s residence). We observed disparities in willingness according to race, comparing Black to White participants, but not when comparing White to American Indian, Alaska Native, Asian, Native Hawaiian, or Pacific Islander participants nor Hispanic to non-Hispanic participants. The disparities in willingness between Black and White participants persisted until we introduced the variables of environmental health literacy, when the difference between these groups was no longer significant in the final regression models. The findings suggest that focusing on environmental health literacy could bridge a gap in willingness to protect oneself based on factors such as race/ethnicity and socioeconomic status, which have been identified in the environmental health literature as resulting in health disparities.

## 1. Introduction

Environmental health literacy (EHL) is a relatively new framework, with its roots in health, science, and environmental literacies, as well as in risk communication and environmental health sciences [1,2]. In the past, the Society for Public Health Education describes EHL as using a combination of environmental literacy and health literacy to seek out and use information to reduce adverse health outcomes [1]. A key tenet of EHL, both in scholarship and in practice, is its emphasis on action—changing behaviors to reduce, mitigate, or avoid exposure to harmful environmental agents—which can ultimately lead to positive health outcomes at both the individual and community level [1,2].

Existing EHL literature emphasizes three pressing ideas. First, improved public health outcomes rely on people making changes in behavior, from using a water filter at home to moving to an entirely different community. In general, when individuals change their behaviors related to environmental exposures, it can have positive public health impacts at the community level [3]. Second, public health researchers and practitioners need to understand the conditions that motivate people to modify their behaviors and take health protective actions [2]. Third, research in this area needs to develop both generalizable ways of measuring EHL in quantitative research [4] and maintain nuances that acknowledge the environmental health context [5].

As reviews of EHL research by Gray [6] and Finn and O’Fallon [1] suggest, there is less certainty in the literature of *how* environmental literacy can be harnessed, deployed, measured, or observed to have a concrete impact on—e.g., reducing or even eliminating—environmental health disparities. The aim of this research study is to integrate these pressing EHL ideas into a quantitative model that can be generalized to adult populations (in our case, residents of North Carolina, who faced two public health challenges in 2020) while acknowledging the key roles of efficacy and specific health contexts. Thus, we approach the topic of environmental health literacy and health disparities to investigate *how* and *for whom* EHL makes a difference in motivating protective actions in the context of two very different environmental health threats.

### 1.1. Environmental Health Literacy

Environmental health literacy refers to a range of skills required to seek out information, then understand, evaluate, and use it to make informed changes [7]. Gray [6] conceptualizes EHL as hierarchical concentric circles, with increased knowledge and skills on the bottom and community-level actions for improved health at the top. Until recently, there has been no established operationalization of EHL, either as a single or as multiple variables; our study joins the others that we are aware of [2,4] that directly tackle the idea of measurement. We propose a model that integrates environmental health literacy via three separate but complementary concepts drawing from public health, health communication, risk communication, and environmental communication.

For our model, we assume that factual (or content) knowledge about environmental exposures will support one’s decision to engage in protective behaviors. That is, people need to understand how environmental exposures influence health, how to avoid those exposures, and the role that scientific research plays in understanding these connections [2]. Thus, factual knowledge is one resource, which we identify as a “knowing the facts” dimension of EHL. Whereas other research tends to rely solely on measures of knowledge [2], we explore two additional concepts. One is a more subjective definition of knowledge. We assume that people need knowledge to make decisions but that there is no universal level of sufficient knowledge. Knowledge sufficiency, a concept from the risk communication literature, reflects the difference in how much a person feels she knows about a health threat (current knowledge) and how much she feels she needs to know to make a good decision (sufficiency threshold) [8]. We consider knowledge sufficiency as a “feeling ready to decide” dimension of EHL. A final concept is efficacy. Once people feel that they have sufficient information and skills to modify their behavior, they need to believe that their actions will protect them [9]. Research has borne this idea out, for example in the relationship between efficacy and motivation to adopt behaviors in the context of the H1N1 influenza pandemic [10]. From the health communication literature, this idea of response efficacy [11,12]—belief in the effectiveness of a responsive behavior—constitutes our “believing it works” dimension of EHL.

In our view, incorporating these three dimensions of knowing, feeling, and believing reflects existing conceptual definitions of EHL and allows us to investigate how EHL might be related to improved health outcomes. We propose to investigate such outcomes in terms of known factors associated with environmental health disparities.

### 1.2. Identified Factors Underlying Environmental Health Disparities

Across the United States, there are consistent disparities in rates of infectious diseases, cancers, cardiovascular diseases, and other serious health outcomes, many of which can be at least partially linked to environmental exposures [13]. Americans from marginalized racial and ethnic groups and those with lower socioeconomic status (SES) experience the highest rates of adverse health outcomes [13,14]. We focus on these two broad categories of people.

In the U.S., marginalized racial and ethnic communities are more likely to have inadequate housing, lower quality education, an unclean environment, and negative health outcomes, even when controlling for personal characteristics [15,16,17]. Exposure to pollution and environmental contaminants is higher among these marginalized groups, highlighting continued instances of environmental injustice [14]. Research has shown that race and ethnicity are related to a community’s obesity rates, exposure to air pollution, lack of access to green spaces, and lower overall life satisfaction [14,15,18,19].

We also consider the role of socioeconomic status, encompassing education, income, and home ownership. The U.S. has above-average rates of poverty and disposable income inequality compared to other developed countries [16]. Homeownership historically represents higher levels of SES; it is directly associated with wealth accumulation, community building, neighborhood stability, and overall economic well-being [20,21]. In contrast, lower neighborhood-level SES is associated with increased psychosocial stress, fewer environmental amenities, and decreased access to parks, high-speed internet, libraries, and other local infrastructure [14,22]. These resources allow people to address potential health risks, and people without access tend to have poorer health [17].

In sum, the same environmental exposures can have very disparate health effects that vary by race, socioeconomic status, and geography [23], leading us to two questions. First, how do race, ethnicity, and SES relate to one’s willingness to engage in protective behaviors against environmental health threats? Second, how might EHL play a role in that relationship?

### 1.3. Two Recently Identified Health Threats in a Social and Cultural Context

Our study addresses these two questions by focusing on two different health threats—infectious disease and industrial contamination—in a single geographic area. We treat the two threats as representative types; that is, these are threats featuring certain characteristics that could be generalized beyond the context of this research study.

#### 1.3.1. Infectious Disease: SARS-CoV-2 and COVID-19

In the last twenty years, the world has dealt with multiple infectious disease outbreaks, including severe acute respiratory syndrome (SARS) in 2003, Middle East respiratory syndrome (MERS) in 2012, and Ebola virus disease (EVD) in 2014 to 2016 [24]. COVID-19 was first recognized in late 2019 in China, showing structural similarities to SARS [25]. Like other disease outbreaks, the virus that causes COVID-19 (SARS-CoV-2) is spread through respiratory transmission, but it is more contagious than SARS and has more variability in symptoms compared to influenza or SARS [26]. Within weeks, the virus had spread across the world.

By September 2020, data showed that COVID-19 was not affecting all U.S. residents equally; there were obvious disparities in the disease’s impacts among Black, Hispanic, and immigrant populations in the United States [27]. Although these marginalized groups may have recognized COVID-19 as a threat, systemic factors beyond their control may have precluded some from properly engaging in preventative behaviors and may have led to this race-based death discrepancy [28]. COVID-19 disease prevention relies on collective actions—such as social distancing, mask wearing, and vaccinations—that might not be individually desirable but are beneficial overall for public health [29]. The morbidity, mortality, economic, and societal impacts of COVID-19 may not affect all Americans equally, but they are equally capable of taking actions to reduce the spread of the disease for everyone.

#### 1.3.2. Industrial Contamination: Per- and Polyfluoroalkyl Substances (PFAS)

A class of thousands of chemicals, per- and polyfluoroalkyl substances (PFAS) are used to make non-stick, water-repellent, and stain-proof products, as well as plastics and firefighting foams [30]. There is no information on the exact number of substances in production, there is no federal agency keeping track of PFAS, and there is limited information on long-term health effects from PFAS exposure [31]. While these substances have been found in people, animals, and drinking water around the world, they receive relatively little mainstream media attention [31] despite their negative effects on health (one study reported 28% of residents in communities contaminated with PFAS developed at least one health condition [32]).

One recent and vivid example comes from North Carolina (NC), a state on the coast of the Atlantic Ocean in the southeastern U.S. Scientists recently discovered that a PFAS manufacturer in Fayetteville, NC, had been discharging wastewater containing PFAS into the Cape Fear River since 1980 [33]. In 2016, PFAS were first detected in the river, which provides drinking water to more than 200,000 residents; the substances were then found in treated drinking water in eastern North Carolina in 2017 and most recently in biological samples of residents in 2017 and 2018 [33]. Because of their ecological persistence in the environment [30], PFAS cannot be remediated in the same way as some other industrial contaminants. The only way to avoid exposure is to buy bottled water, use in-home filtration systems, or move to a different community.

#### 1.3.3. Social and Cultural Context

Within the United States, we focus on North Carolina as a social and cultural context. The state is geographically varied with identifiable spatial patterns of racially and economically segregated communities [18]. The state’s demographics are diverse, with Hispanic people representing 10% of the state’s population, and Black people making up 22% (compared to 13% of the United States’ population) [34]. The environmental justice (EJ) movement has its roots in Warren County, North Carolina, where the siting of a hazardous waste landfill in the 1980s spurred a national conversation on inequitable and unjust exposure to environmental contaminants [14]. Even today, adverse health outcomes related to air pollution are highest in the state’s Black and low SES communities [18]. Predominantly Black communities in North Carolina experience lower rates of municipal water access, higher rates of contaminated drinking water, and more water-related illnesses that require emergency department visits [34,35]. Additionally, low-income and marginalized racial and ethnic communities have disproportionately higher rates of obesity, as compared to the state’s White residents [36].

In 2020, North Carolinians faced both the COVID-19 pandemic and the presence of per- and polyfluoroalkyl substances (PFAS) in drinking water. COVID-19 was an international emergency, receiving almost constant media coverage, and—at least at the outset—represented seemingly equal risk to all people within the state. In contrast, PFAS contamination was, for the most part, a localized problem—certain communities in North Carolina have been impacted while others are not affected at all. Conducting our study in North Carolina also offers a single geographic location, where we can acknowledge the local context of these environmental threats.

### 1.4. Hypotheses and Research Questions

For this study, we investigate the relationships between environmental health literacy (defined as our knowing, feeling, and believing dimensions), common characteristics associated with health disparities (race and ethnicity, socioeconomic status) in terms of willingness to engage in protective behaviors while controlling for other background characteristics. On this basis, we propose a set of hypotheses and research questions.

**Hypothesis** **1a.** *There will be a positive association between the willingness to engage in protective behaviors and environmental health literacy.*

**Hypothesis** **1b.** *There will be a positive association between the willingness to engage in protective behaviors and socioeconomic status (education and income).*

**Hypothesis** **2a.** *There will be a negative difference in the willingness to engage in protective behaviors and marginalized race and ethnicity identity (compared to White and non-Hispanic identity).*

**Hypothesis** **2b.** *There will be a negative difference in the willingness to engage in protective behaviors and renting (compared to owning) one’s residence.*

*Research Question 1:* How does accounting for environmental health literacy impact the association of (a) race/ethnicity and (b) socioeconomic factors with a willingness to engage in protective behaviors? *Research Question 2:* How do the relationships differ between environmental health literacy, willingness to engage in protective behaviors, and (a) race and ethnicity and (b) socioeconomic factors in the context of two very different environmental health threats?

We focus especially on the comparison of these different health threats (RQ2) for a number of reasons. First, both COVID-19 and PFAS exposure put North Carolinians in a position to seek out information about an environmental health issue, make decisions, and modify their behaviors. Second, the context surrounding these threats are very different. Morbidity and mortality rates of COVID-19 have disproportionately impacted marginalized racial and ethnic communities and those with lower socioeconomic status. In contrast, the ability to avoid PFAS in drinking water increases with income levels. Reducing the spread of COVID-19 relies on everyone enacting public health measures, while reducing PFAS exposure requires either large-scale shifts in manufacturing processes or individual changes at home. The different characteristics of these two health threats provide a good basis for comparison.

## 2. Materials and Methods

We collected survey data from 15 to 20 June 2020, at North Carolina State University using the Qualtrics survey platform. NC State University contracted with Dynata, a survey research firm, to reach participants who reside in North Carolina. Dynata recruits diverse participants through programs that offer reward points for the completion of survey questionnaires and other activities. This nonprobability-based sample was not an ideal method for collecting responses, but we could not employ our planned addressed-based mail survey due to the onset of the COVID-19 global pandemic in early 2020. To approximate a representative sample, respondents were recruited using quotas for biological sex, age, race, and ethnicity based on the latest U.S. Census population estimates (from 2019) in the state of North Carolina. The total sample size of verified (through ZIP code) North Carolina residents was 1505; there were two subgroups who received questions either about COVID-19 (*n* = 779) or PFAS (*n* = 726).

In preparing our survey questionnaire, we followed recommendations on structure, wording, and length, based on the most recent published guidance and best practices [37,38]. Exact wording of questions is listed in Table A1 (for dependent variables and environmental health literacy variables) and Table A2 (for all other independent variables). The univariate descriptive statistics for each variable in our regression models can be found in Table A3.

Our dependent variable, willingness to engage in protective behaviors, was measured with two items for each topic of COVID-19 and PFAS. We measured environmental health literacy with three variables: specific factual knowledge, knowledge sufficiency, and response efficacy. Specific factual knowledge was measured with two true and false questions regarding objective facts about each health topic; these were coded with correct responses as 1 and incorrect or blank responses as 0, then summed to form composite variables. Knowledge sufficiency, which emphasizes a more subjective aspect of knowledge and has been employed in the risk and health communication literature [8,39,40,41], was based on two survey items. The first asked participants to rate their current level of knowledge on a scale from 0 to 100, and the second asked them to indicate how much knowledge they felt they would need to make a good decision. Subtracting their desired knowledge from their current knowledge, representing their knowledge sufficiency, indicates a person’s average feeling of having enough information to make an informed decision. Response efficacy corresponded closely to the dependent variable items. We asked participants to indicate how effective each of the behaviors was at preventing exposure to COVID-19 or PFAS. Other independent variables included demographic characteristics such as residing in coastal counties (where PFAS has received more public attention), age, sex, level of education, household income, renting or owning their residence, ethnicity, and race.

For our research, we also must account for other characteristics that might be related to the dependent and independent variables, including people’s beliefs, values, and informational resources. Research in science and environmental communication indicates that these factors play especially outsized roles in shaping people’s view of novel and uncertain topics [42], including the adoption of new behaviors [43]. This literature has explored how political ideology shapes responses to both controversial and non-controversial issues [44]; how religiosity in conjunction with knowledge is associated with different views on nanotechnology [45]; and how trust in various kinds of actors and institutions relates to perceptions of environmental hazards [46]. In addition, of course, one of the biggest contributors to different levels of preparedness and feelings of efficacy might be access to informational resources and the skills needed to put that information into practice. The dynamics of attention to news information have been explored in various health, science, and political communication contexts [47,48,49]. Our final set of independent variables, therefore, includes political ideology, religiosity, and trust in research. Informational resources were measured as attention to news with two variables: attention to science and health news and attention to government and politics news.

For analysis, we employed ordinary least squares regressions [50], an analytic framework common in environmental communication [51], science communication [52,53,54], and health communication research [55]. For each health threat context, we regressed the independent variables on the dependent variable in four different groupings: Block 1 contained demographic variables, Block 2, personal values and beliefs, Block 3, attention to news, and Block 4, environmental health literacy. Model 1 included only Block 1 independent variables and the dependent variable, Model 2 included Blocks 1 and 2 and the dependent variable, and so on. Constructing our models in this manner has two advantages. First, it allows us to see how much certain logical groupings of variables contribute to explaining the variance in the dependent variable. Second, we can compare simpler to more complex models, revealing when—and to some extent how—some variables might be significant in a simpler model but not significant in more comprehensive models.

## 3. Results

Because our analysis comprised separate regression models on two different environmental health topics, we first describe these regression results independently. Following this overview, we compare the model results in more detail.

### 3.1. Regression Model Results: COVID-19

The standardized coefficients from the COVID-19 regression models are displayed in Table 1. Full model results with both unstandardized and standardized coefficients are reported in Table S1. In Model 1, R^2^ = 0.073, *F*(9, 700) = 6.131, *p* < 0.001, we found significant associations between willingness to engage in protective behaviors and age, sex, education, renting a residence, and self-identifying as Black (compared to White). Model 2 resulted in significant incremental change, ΔR^2^ = 0.221, Δ*F*(3, 697) = 72.903, *p* < 0.001; in this model, political ideology and trust in research were associated with willingness. Model 3 had significant incremental change, ΔR^2^ = 0.009, Δ*F*(2, 695) = 4.465, *p* = 0.012, with attention to science and health news as a significant variable. In Model 4, ΔR^2^ = 0.260, Δ*F*(3, 692) = 137.045, *p* < 0.001, all three environmental health literacy variables had significant associations.

Overall, in the final regression model (R^2^ = 56.303%), there were six independent variables that were significantly related to the participants’ willingness to engage in protective behaviors against COVID-19. These were age (β = 0.121, *p* < 0.001), sex (female compared to male participants were more willing; β = 0.068, *p* = 0.014), and trust in research (β = 0.170, *p* < 0.001). Our three variables representing the multidimensional definition of EHL were also significant: COVID-specific factual knowledge (β = 0.128, *p* < 0.001), knowledge sufficiency (β = 0.055, *p* < 0.001), and response efficacy (β = 0.566, *p* < 0.001).

Missing from the significant relationships in Model 4 were variables typically associated with environmental health disparities: race, ethnicity, and socioeconomic status. Some of these variables, along with others, were significant in earlier models but not the final model. Participants self-identifying as Black were more willing than their White counterparts in Models 1 through 3, but not in Model 4. Level of education and renting/owning home were both significant and positive in Model 1 but not in Models 2–4. Attention to science and health news was positively related to willingness in Model 3 but not Model 4. Political ideology was negatively related to willingness in a consistent way in Models 2 and 3 but not in Model 4.

### 3.2. Regression Model Results: Per- and Polyfluoroalkyl Substances (PFAS)

The results of our regression models predicting willingness to engage in PFAS-protective behaviors are shown in Table 2 (full model results with both unstandardized and standardized coefficients are reported in Table S2). In Model 1, R^2^ = 0.087, *F*(9, 691) = 7.354, *p* < 0.001, we found significant associations between a willingness to engage in protective behaviors and age, sex, and self-identifying as Black (compared to White). Subsequent models resulted in significant incremental changes. In Model 2, ΔR^2^ = 0.049, Δ*F*(3, 688) = 12.940, *p* < 0.001, religiosity and trust in research were associated with willingness. Model 3, ΔR^2^ = 0.036, Δ*F*(2, 686) = 14.908, *p* < 0.001, attention to science and health news was significant. In Model 4, ΔR^2^ = 0.215, Δ*F*(3, 683) = 80.601, *p* < 0.001, PFAS-specific factual knowledge and response efficacy were significant variables.

Overall, the final PFAS regression model (R^2^ = 38.859%) revealed four variables that had significant associations. Female respondents (β = 0.083, *p* = 0.009) and those more trusting in research (β = 0.103, *p* = 0.002) were more willing to engage in protective behaviors. Two of the three dimensions of EHL, PFAS-specific factual knowledge (β = 0.075, *p* = 0.015) and response efficacy (β = 0.503, *p* < 0.001), were also associated with the dependent variable. Similar to the COVID-19 regressions, some variables were significant in earlier models but not the final model. These were identifying as Black (Models 1–3), religiosity (Model 2 only), and attention to science and health news (Model 3 only).

### 3.3. Comparison of COVID-19 and PFAS Regression Results

Directly comparing the final regression models for COVID-19 and PFAS (see Table 3), the results provide support for Hypothesis 1a: Two out of three EHL dimensions were positively related to a willingness to engage in protective behaviors. We did not find support for Hypothesis 1b (education, income, and willingness), 2a (negative difference in willingness for marginalized racial or ethnic groups), or 2b (negative difference for renters vs. owners). To answer Research Question 1, focusing on differences between health contexts, we found that factual knowledge and response efficacy had important and consistent associations with willingness to engage in protective behaviors, whereas knowledge sufficiency was significant only in the COVID-19 context. Finally, to the answer to Research Question 2 (how EHL impacts relationships between racial/ethnic categories, socioeconomic status, and willingness), the evidence shows that accounting for EHL eliminates small but important differences in willingness to engage in these behaviors between Black and White respondents in both environmental health contexts.

## 4. Discussion

In this study, we investigated the willingness to protect oneself from health threats through the lenses of environmental health literacy and health communication. Our research provides an intriguing comparison of two different kinds of environmental health threats. COVID-19, one could say, represents a health threat that is widespread, has a high level of public awareness (only 3% of respondents reported not having heard about COVID-19 before participating in our survey), and is debated in local, state and national contexts. In contrast, PFAS contamination in North Carolina is more specific in geography and community, has generally low public awareness (59.1% of respondents had not heard of PFAS before completing our survey), and is discussed most prominently at the local level. One might ask, therefore, how are people in North Carolina responding to these very different health threats?

Our results suggest an answer. In the final models (see Table 3), we found clear consistencies: significant roles for sex (women generally are more risk averse than men), trust in research (who are more receptive to public health recommendations), and environmental health literacy. When they differed, with variables such as age and political ideology having significant associations for COVID-19 responses, those differences can be at least partially explained by the context of the global pandemic in the United States. After all, COVID-19 has affected older people more severely and has become a highly politically charged topic. Among the environmental health literacy variables, knowledge sufficiency was significant for COVID-19 but not for PFAS. This disparity could also be explained through the contexts: As one moves along the insufficiency–sufficiency continuum, the more confident a person can be that they can make a good health decision. Without high public awareness (and a resulting increased circulation of information about protective behaviors), as well as the uncertainty associated with the health effects of PFAS, the feeling of having enough information seems not to be enough to result in higher levels of willingness to adopt proactive behaviors.

These results contribute to the growing literature on environmental health literacy in three ways. First, the study of EHL will benefit from integrating concepts from other areas of research, such as health and risk communication. Second, health disparities between groups of people who vary widely in their access to public health resources might be reduced, in part, by increasing environmental health literacy in those individuals. Third, by comparing our models in the context of two different environmental health threats, our research indicates key similarities and differences in how people respond to environmental health threats of different kinds. Prior to discussing these contributions in more detail, we acknowledge some shortcomings of the study.

### 4.1. Limitations

Our research is not without limitations, and we address three of them. The first limitation concerns our sampling procedure. While we had intended to conduct a robust mail survey using a probability sample, those plans were scrapped in early 2020 due to the onset of the COVID-19 global pandemic. As a result, we weighed our options and decided to contract with Dynata, using a convenience sample with quotas reflecting the U.S. Census estimated from 2019. Even with these quotas, we cannot completely rule out bias in our data due to sampling error. The second limitation pertains to our variables reflecting how people get information. For this study, we focused primarily on topics of news and information. The potentially different roles for online versus traditional (print and broadcast) news sources cannot be examined with the data we collected. To maintain a narrower theoretical focus, we also chose not to include measures of interpersonal discussion, which can be another channel for information about health and risk. Given ongoing nationwide discussions of social media and interpersonal communication networks, the role they play in shaping environmental health concerns merits further investigation. Finally, our survey data were collected at a single point in time, and we approach any suggestion of causal relationship judiciously and with appropriate caution. Our main contributions are described with this caveat in mind.

### 4.2. Contributions

A starting point for our research was incorporating existing concepts and measures from public health and communication disciplines in a study of environmental health literacy. In the regression model results, our three dimensions of EHL (factual knowledge, knowledge sufficiency, and response efficacy) were associated with the willingness to engage in protective behaviors in different but complementary ways. Factual knowledge has received an abundance of attention in EHL research [2,4,6]. Not surprisingly, response efficacy had the strongest association with the dependent variable; believing in corrective actions against health threats is often a strong contributor to self-efficacy, behavioral intentions, and ultimately, behaviors [10,12]. A novel contribution of our models is the unique role for knowledge sufficiency, which aligns with other EHL studies that highlight variables other than factual knowledge (which is sometimes assumed to be sufficient for literacy) [5]. Among our respondents, feeling that they had enough information to make a good decision (for themselves) was indeed related to their willingness to protect themselves against COVID-19.

We would argue that the “feeling ready to decide” dimension deserves more focus in the EHL scholarship. One could interpret it, at least tentatively, as an important complement to both factual knowledge and response efficacy; a crucial step between knowing the facts and believing in protective behaviors may be feeling adequately comfortable and confident in making a decision. Of course, we must acknowledge that knowledge sufficiency did not play a significant role in the context of PFAS, which could be due to several reasons such as its novel and geographically limited impact on North Carolina residents. This echoes findings from Irvin and colleagues [56], who noted that sufficient knowledge of well water contamination and ownership was not related to health protective behaviors with their water. Nevertheless, it certainly follows from the health communication literature that feeling uncertain about a health threat (i.e., not feeling like one knows enough yet) is not a necessary precursor to acting and preserving such uncertainty can be advantageous in certain health contexts [8,57]. Future EHL research could provide a fuller understanding of when knowledge sufficiency makes a difference in decision-making.

The second focus in our research study was on health disparities and some commonly understood associations or even causes of those disparities. For simplicity, we focused on two broad areas: marginalized racial and ethnic groups and socioeconomic status (SES). In the very first steps of our regression models, education was the only aspect of SES that was associated with willingness. In later models, when we accounted for other characteristics of respondents, the significance of education diminished. Similar results appeared for Gray and colleagues [5] when testing an EHL evaluation tool, where education was initially significantly related to EHL in the overall sample but was not after analyzing the groups of participants separately. Overall, our models showed little support for SES contributing to people’s willingness to engage in protective behaviors. This was a bit surprising; other EHL literature has shown that lower SES participants are motivated to learn about their exposures and take action [58]. That study employed a very different research design (a direct intervention among low-income housing residents) from ours, and differing results suggest more research is needed on the roles of socioeconomic factors.

We did observe differences in willingness for certain racial categories in our models, and these differences persisted until the final model when we introduced our environmental health literacy variables. We focused on these categories of people, encompassing major racial and ethnic groups identified in previous research, with the expectation that disparities in willingness would be akin to disparities in health outcomes. Our observed results were, in fact, the opposite. While Black participants expressed a level of willingness significantly different from White participants, the difference emerged because Black respondents were *much more willing* than their White counterparts. We theorize two reasons for this difference, although we leave open the possibility for other explanations.

First, one could interpret these results as indicative of an increased feeling of susceptibility among the Black residents of North Carolina to these health threats. For COVID-19, increased susceptibility among marginalized racial and ethnic groups has been apparent in public health data throughout the pandemic. If individuals are feeling more susceptible, then it stands to reason they also become more willing to protect themselves. Similarly, PFAS contamination has been associated with environmental injustice in affected communities, and a link from susceptibility to willingness to protect oneself might be expected. A second interpretation may be less psychological, instead pointing toward key sociological differences between communities and groups. Given the history of environmental injustice in states like North Carolina, where marginalized racial and ethnic communities have, on balance, suffered more exposure to environmental threats, there may be systemic reasons for the disparity in willingness that we observed. For example, if these groups of people are less served by government agencies or green infrastructure development [59], they may feel that it is up to their own communities to defend themselves against environmental health threats.

Our final contribution with this research is in comparing two types of environmental health threats people face: one based on human-to-human infectious disease that affects entire communities, regions, and countries and where individual actions (such as wearing masks) can have demonstrable protective effects (COVID-19); and one based on widespread industrial contamination affecting narrower geographic areas and where individual actions can help but full protection requires governmental agency action, lawsuits, and corporate responsibility (PFAS). Having such different threats as the foci of our study leads us to some tentative conclusions across environmental health contexts. EHL seems to mitigate disparities between groups of people (in essence, getting different groups “on board” for taking action). Women tend to be more risk averse [60] and therefore more willing to take action. Furthermore, trust in research maintains a direct association with willingness to protective oneself. Our final models (see Table 3) demonstrated notable consistency in these relationships. We feel confident in contributing these findings to the generalizable knowledge in environmental health literacy.

## 5. Conclusions

With this research study, we aimed to investigate how environmental literacy can be harnessed, deployed, measured, or observed to have a concrete impact on—e.g., reducing or even eliminating—environmental health disparities. Our study made several modest contributions toward a better understanding of environmental health literacy and its potential for positive impacts on people’s willingness to combat environmental health threats. We focused on bridging different disciplines—environmental health sciences, health and risk communication, and public health—to develop and model the knowing, feeling, and believing dimensions of EHL. Across two different contexts, we observed strong associations between EHL and a willingness to engage in protective behaviors, and we identified some other commonalities. The promotion of EHL, with a more expansive definition that goes beyond factual knowledge (as others have argued [6]), seems like a worthwhile path forward in both the research and practice of communicating environmental health threats to those who face them in their everyday lives.

## Figures and Tables

**Table 1:** Results of stepwise multiple regression predicting willingness to engage in protective behaviors against COVID-19 based on demographics, values/beliefs, news use, and environmental health literacy. *N* = 716 ^1^.

	Model 1	Model 2	Model 3	Model 4
**Block 1: Demographics**				
Coastal counties	−0.061	−0.058	−0.061	−0.035
Age	0.228 ***	0.264 ***	0.258 ***	0.121 ***
Sex (female)	0.090 *	0.103 **	0.114 ***	0.068 *
Education	0.082 *	0.016	0.011	0.015
Income	0.076	0.058	0.044	0.029
Rent home	0.112 **	0.052	0.049	0.037
Ethnicity (Hispanic)	−0.013	−0.035	−0.035	−0.016
Race (Black)	0.089 *	0.086 *	0.075 *	0.037
Race (American Indian, Alaska Native, Asian, Native Hawaiian, Pacific Islander, or other)	0.015	0.021	0.006	−0.008
Block R^2^ (%)	7.307%			
**Block 2: Values and Beliefs**				
Political ideology		−0.137 ***	−0.128 ***	−0.057
Religiosity		0.043	0.029	−0.015
Trust in research		0.429 ***	0.409 ***	0.170 ***
Block R^2^ (%)		22.139%		
**Block 3: Attention to News**				
Science and health news			0.132 *	0.075
Politics and government news			−0.040	−0.063
Block R^2^ (%)			0.895%	
**Block 4: Environmental Health Literacy**				
Factual knowledge				0.128 ***
Knowledge sufficiency				0.055 *
Response efficacy				0.566 ***
Block R^2^ (%)				25.962%
**Final Model R^2^(%)**				**56.303%**

^1^ Cell entries are standardized regression coefficients. *N* = 716. * *p* < 0.05, ** *p* < 0.01, *** *p* < 0.001.

**Table 2:** Results of stepwise multiple regression predicting willingness to engage in protective behaviors against PFAS based on demographics, values/beliefs, news use, and environmental health literacy. *N* = 706 ^2^.

	Model 1	Model 2	Model 3	Model 4
**Block 1: Demographics**				
Coastal counties	0.070	0.090 *	0.099 **	0.040
Age	−0.129 **	−0.129 **	−0.130 **	−0.036
Sex (female)	0.082 *	0.071	0.110 **	0.083 **
Education	−0.004	−0.030	−0.061	−0.062
Income	−0.040	−0.049	−0.056	−0.048
Rent home	0.014	−0.001	0.004	−0.003
Ethnicity (Hispanic)	0.047	0.033	0.025	0.045
Race (Black)	0.145 ***	0.141 ***	0.122 **	0.060
Race (American Indian, Alaska Native, Asian, Native Hawaiian, Pacific Islander, or other)	−0.021	−0.002	0.005	0.019
Block R^2^ (%)	8.741%			
**Block 2: Values and Beliefs**				
Political ideology		−0.025	−0.009	−0.021
Religiosity		0.075 *	0.065	0.045
Trust in research		0.210 ***	0.154 ***	0.103 **
Block R^2^ (%)		4.874%		
**Block 3: Attention to News**				
Science and health news			0.129 *	0.033
Politics and government news			0.089	0.099
Block R^2^ (%)			3.598%	
**Block 4: Environmental Health Literacy**				
Specific factual knowledge				0.075 *
Knowledge sufficiency				−0.048
Response efficacy				0.503 ***
Block R^2^(%)				21.646%
**Final Model R^2^(%)**				**38.859%**

^2^ Cell entries are standardized regression coefficients. *N* = 706. * *p* < 0.05, ** *p* < 0.01, *** *p* < 0.001.

**Table 3:** Results of final multiple regressions ^3^ predicting willingness to engage in protective behaviors against COVID-19 and PFAS based on demographics, values/beliefs, news use, and environmental health literacy.

	COVID-19	PFAS
**Block 1: Demographics**		
Coastal counties	−0.035	0.040
Age	0.121 ***	−0.036
Sex (female)	0.068 *	0.083 **
Education	0.015	−0.062
Income	0.029	−0.048
Rent home	0.037	−0.003
Ethnicity (Hispanic)	−0.016	0.045
Race (Black)	0.037	0.060
Asian, Pacific Islander, or Native American	−0.008	0.019
Block R^2^ (%)	7.307%	8.741%
**Block 2: Values and Beliefs**		
Political ideology	−0.057	−0.021
Religiosity	−0.015	0.045
Trust in research	0.170 ***	0.103 **
Block R^2^ (%)	22.139%	4.874%
**Block 3: Attention to News**		
Science and health news	0.075	0.033
Politic and government news	−0.063	0.099
Block R^2^ (%)	0.895%	3.598%
**Block 4: Environmental Health Literacy**		
Specific factual knowledge	0.128 ***	0.075 *
Knowledge sufficiency	0.055 *	−0.048
Response efficacy	0.566 ***	0.503 ***
*Block R^2^ (%)*	25.962%	21.646%
**Final Model R^2^(%)**	56.303%	**38.859%**

^3^ Cell entries are standardized regression coefficients. *N* = 716 (COVID-19) and 706 (PFAS). * *p* < 0.05, ** *p* < 0.01, *** *p* < 0.001.

## Data Availability

The data presented in this study are available on request from the corresponding author. The data are not publicly available due to the collection of identifying information (ZIP codes together with specific demographic information) from survey respondents.

## Supplementary Materials

The following are available online at https://www.mdpi.com/article/10.3390/ijerph19052701/s1, Table S1: Full regression model results for COVID-19 with both unstandardized and standardized coefficients; Table S2: Full regression model results for PFAS with both unstandardized and standardized coefficients.

## Appendix A

**Table A1:** Survey item wording for dependent variable and environmental health literacy variables.

Dependent Variable	Response Options
Willingness to engage in protective behaviors: “How willing are you to perform each of these behaviors to prevent your exposure to [COVID-19/PFAS]?”	
“Social distancing,” or having space between you and other people ^1^.	Not at all willing to Extremely willing (5 options)
Wearing a mask in public ^1^.	
Drinking bottled water rather than tap water ^2^.	
Removing furniture and carpet with stain resistant treatments from my home ^2^.	
**Environmental Health Literacy**	
Specific factual knowledge: “Can you tell us if each of the following statements is true or false, or if you don’t know?”	
The coronavirus that causes COVID-19 was first identified in Wuhan, China ^1^.	True, False, Don’t Know
COVID-19 is a fatal health threat only to people over the age of 60.^1^	
PFAS are substances that do not exist naturally and are only man-made ^2^.	
All PFAS chemicals (including PFOS, PFOA, GenX) act the same way in humans and the environment ^2^.	
Knowledge sufficiency: “We would like you to rate your knowledge about the risks of [COVID-19/PFAS]. Please use a scale of 0 to 100, where 0 means knowing nothing and 100 means knowing everything you could possibly know about this topic.”	
Using this scale, how much do you think you currently know about the risk from [COVID-19/PFAS]?	Open-ended response from 0 to 100
Using the same scale of 0 to 100, how much information about [COVID-19/PFAS] would be sufficient for you, that is, good enough for your purposes?	
Response efficacy: “As with many threats to health, there are different behaviors people might engage in to prevent exposure to viruses like COVID-19. How effective do you think each of the following activities is at preventing infection?”	
“Social distancing,” or having space between you and other people ^1^.	Not effective at all to Very effective (5 options)
Wearing a mask in public ^1^.	
Drinking bottled water rather than tap water ^2^.	
Removing furniture and carpet with stain resistant treatments from my home ^2^.	

^1^ Asked only in the group of respondents receiving COVID-19 questions. ^2^ Asked only in the PFAS group.

**Table A2:** Survey item wording for all other independent variables.

Demographic Variables	Response Options
Coastal counties: “What is your ZIP code?”	Open-ended, then coded for two regions (“Coastal” and “Non-coastal”
Age: “What year were you born?”	Drop-down list of years, then recoded by subtracting birth year from 2020
Sex: “What is your sex?”	Male, Female
Education: “What is the highest level of schooling you have completed?”	Less than high school to Doctorate (Ph.D.) (8 options)
Income: “In 2019, was your household income before taxes…”	Less than USD 10,000, to More than USD 150,000 (12 options)
Rent home: “Do you rent or own your current residence?”	Rent, Own, Not Sure
Ethnicity: “What is your ethnicity?”	Hispanic, Not Hispanic
Race: “What is your race? (Select all that apply.)”	White, Black or African American, American Indian or Alaska Native, Asian, Native Hawaiian or Pacific Islander, other
**Values and Beliefs**	
Political ideology: “The terms ‘liberal’ and ‘conservative’ may mean different things to different people depending on the kind of issue one is considering. How would you describe your views in terms of…?”	
Economic issues	Very liberal to very conservative (5 options)
Social issue	
Religiosity: “How much guidance does religion play in your everyday life?”	No guidance at all to A lot of guidance (5 options)
Trust in research: “How much do you trust the following sources of information to tell you the truth about health risks facing your community?”	
Scientists at universities	Do not trust at all to Trust completely (5 options)
North Carolina Department of Environmental Quality	
North Carolina Department of Health & Human Services	
**Attention to News**	
“When you [read a newspaper or a news website/watch television news], how much attention do you pay to news about …?”	
Science and technology	None (including “no exposure”) to A lot (5 options)
Health and medicine	
Government and Politics	

**Table A3:** Univariate descriptive statistics for independent and dependent variables.

	COVID-19			PFAS		
	M/% ^1^	SD ^2^	Reliability ^3^	M/% ^1^	SD ^2^	Reliability ^3^
**Dependent Variable**						
Willingness to engage in protective behaviors	4.080	1.046	*r* = 0.662	3.121	1.129	*r* = 0.444
**Environmental Health Literacy**						
Specific factual knowledge	1.666	0.591	--	0.770	0.719	--
Knowledge sufficiency	4.740	26.521	--	−31.610	31.422	--
Response efficacy	3.681	1.042	*r* = 0.668	2.753	0.973	*r* = 0.358
**Demographics**						
Coastal counties	27.785%	--	--	27.493%	--	--
Age	47.034	17.870	--	46.366	17.435	--
Sex (female)	51.319%	--	--	53.243%	--	--
Education	4.125	1.608	--	4.189	1.627	--
Income	6.536	3.495	--	6.766	3.549	--
Rent home	28.740%	--	--	30.853%	--	--
Ethnicity (Hispanic)	8.753%	--	--	8.943%	--	--
Race (Black)	20.740%	--	--	20.270%	--	--
Race (American Indian, Alaska Native, Asian, Native Hawaiian, Pacific Islander, or other)	10.700%	--	--	10.676%	--	--
**Values and Beliefs**						
Political ideology	3.031	1.131	*r* = 0.724	3.007	1.135	*r* = 0.727
Religiosity	3.039	1.340	--	2.988	1.327	--
Trust in research	3.317	0.920	α = 0.833	3.307	0.919	α = 0.840
**Attention to News**						
Science and health news	2.685	1.266	α = 0.853	2.701	1.238	α = 0.843
Politics and government news	2.933	1.450	*r* = 0.569	2.937	1.380	*r* = 0.507

^1^ For continuous variables we report the arithmetic mean. For categorical variables (e.g., sex), we report the percentage of respondents in that category. ^2^ For continuous variables only, we report the standard deviation. ^3^ For composite variables, we provide a suitable indicator of reliability: Pearson’s *r* for variables made up of two items (all *p* < 0.001), and Cronbach’s alpha (α) for variables made up of three or more items. This table reports the total number of participants who received each question. Due to the presence of missing values on some measures, the number of participants included in the regression models was *N* = 716 for COVID-19 and *N* = 706 for PFAS.
