# Supplementary material for: Environmental Health Literacy as Knowing, Feeling, and Believing: Analyzing Linkages between Race, Ethnicity, and Socioeconomic Status and Willingness to Engage in Protective Behaviors against Health Threats
Source: Int J Environ Res Public Health. 2022 Feb 25;19(5):2701. doi: 10.3390/ijerph19052701 (PMC8910584; doi:10.3390/ijerph19052701)
Supplement: Supplementary file 1 [file ijerph-19-02701-s001.zip › ijerph-1534644-supplementary.pdf]

Table S1: Full regression model results for COVID-19 with both unstandardized and standardized coefficients

	Model 1		Model 2		Model 3		Model 4	
	B (S.E.)	β	B (S.E.)	β	B (S.E.)	β	B (S.E.)	β
Intercept	2.926 (0.181)		1.739 (.233)		1.677 (0.234)		0.615 (0.194)	
Block 1: Demographics								
Coastal Counties	−0.142 (0.086)	−0.061	−0.135 (0.075)	−0.058	−0.143 (0.075)	−0.061	−0.081 (0.06)	−0.035
Age	0.013 (0.002)	0.228 ***	0.015 (0.002)	0.264***	0.015 (0.002)	0.258 ***	0.007 (0.002)	0.121 ***
Sex (female)	0.187 (0.08)	0.090 *	0.214 (0.071)	0.103**	0.236 (0.071)	0.114***	0.140 (0.057)	0.068*
Education	0.053 (0.027)	0.082 *	0.011 (0.024)	0.016	0.007 (0.024)	0.011	0.010 (0.019)	0.015
Income	0.023 (0.013)	0.076	0.017 (0.012)	0.058	0.013 (0.012)	0.044	0.009 (0.009)	0.029
Rent home	0.257 (0.092)	0.112 **	0.12 (0.081)	0.052	0.113 (0.081)	0.049	0.085 (0.064)	0.037
Ethnicity (Hispanic)	−0.049 (0.148)	−0.013	−0.131 (0.13)	−0.035	−0.132 (0.129)	−0.035	−0.059 (0.103)	−0.016
Race (Black)	0.228 (0.108)	0.089 *	0.221 (0.096)	0.086*	0.193 (0.096)	0.075 *	0.095 (0.077)	0.037
Race (American Indian, Alaska Native, Asian, Native Hawaiian, Pacific Islander, or Other)	0.049 (0.138)	0.015	0.069 (0.121)	0.021	0.019 (0.122)	0.006	−0.026 (0.097)	−0.008
Block R^2 (%)		*7.307%*						
Block 2: Values and Beliefs								
Political ideology			−0.126 (.034)	−0.137 ***	−0.118 (0.034)	−0.128 ***	−0.053 (0.027)	−0.057
Religiosity			0.033 (.026)	0.043	0.023 (0.026)	0.029	−0.011 (0.021)	−0.015
Trust in Research			0.483 (.038)	0.429 ***	0.460 (0.038)	0.409 ***	0.191 (0.033)	0.170 ***
Block R^2 (%)				*22.139%*				
Block 3: Attention to News								
Science & health news					0.109 (.048)	0.132 *	0.062 (0.038)	0.075
Politics & government news					−0.029 (.041)	−0.040	−0.045 (0.033)	−0.063
Block R^2 (%)						*0.895%*		
Block 4: Environmental Health Literacy								
Factual knowledge							0.231 (0.049)	0.128 ***
Knowledge sufficiency							0.002 (0.001)	0.055 *
Response efficacy							0.567 (0.029)	0.566 ***

Block R^2(%)								*25.962%*
Final Model R^2(%)								***56.303%***
N = 716								

Table S2: Full regression model results for PFAS with both unstandardized and standardized coefficients

	**Model 1**		**Model 2**		**Model 3**		**Model 4**	
	B (S.E.)	β	B (S.E.)	β	B (S.E.)	β	B (S.E.)	β
Intercept	3.347 (0.205)		2.486 (.288)		2.229 (0.287)		0.79 (0.264)	
Block 1: Demographics								
Coastal Counties	0.175 (0.092)	0.070	0.225 (0.09)	0.090 *	0.249 (0.088)	0.099 **	0.101 (0.077)	0.040
Age	−0.008 (0.003)	−0.129 **	−0.008 (0.003)	−0.129 **	−0.008 (0.003)	−0.130 **	−0.002 (0.002)	−0.036
Sex (female)	0.185 (0.084)	0.082 *	0.159 (0.082)	0.071	0.248 (0.082)	0.110 **	0.187 (0.071)	0.083 **
Education	−0.003 (0.03)	−0.004	−0.021 (0.029)	−0.030	−0.043 (0.029)	−0.061	−0.043 (0.025)	−0.062
Income	−0.013 (0.014)	−0.040	−0.015 (0.014)	−0.049	−0.018 (0.014)	−0.056	−0.015 (0.012)	−0.048
Rent home	0.034 (0.098)	0.014	−0.003 (0.097)	−0.001	0.01 (0.095)	0.004	−0.008 (0.082)	−0.003
Ethnicity (Hispanic)	0.187 (0.157)	0.047	0.134 (0.154)	0.033	0.102 (0.151)	0.025	0.183 (0.13)	0.045
Race (Black)	0.407 (0.113)	0.145 ***	0.395 (0.112)	0.141 ***	0.34 (0.111)	0.122 **	0.169 (0.096)	0.060
Race (American Indian, Alaska Native, Asian, Native Hawaiian, Pacific Islander, or Other)	−0.078 (0.146)	−0.021	−0.006 (0.144)	−0.002	0.02 (0.141)	0.005	0.07 (0.122)	0.019
Block R^2 (%)		*8.741%*						
Block 2: Values and Beliefs								
Political ideology			−0.025 (0.04)	−0.025	−0.009 (0.039)	−0.009	−0.021 (0.034)	−0.021
Religiosity			0.064 (0.032)	0.075 *	0.055 (0.032)	0.065	0.038 (0.028)	0.045
Trust in Research			0.257 (0.045)	0.210 ***	0.188 (0.046)	0.154***	0.126 (0.04)	0.103 **
Block R^2 (%)				*4.874%*				
Block 3: News Use								
Science & health news					0.117 (.058)	0.129 *	0.03 (0.051)	0.033
Politics & government news					0.072 (.051)	0.089	0.08 (0.044)	0.099

Block R^2 (%)	*3.598%*		
Block 4: Environmental Health Literacy			
Specific factual knowledge		0.117 (0.048)	0.075 *
Knowledge sufficiency		−0.002 (0.001)	−0.048
Response efficacy		0.588 (0.038)	0.503 ***
Block R^2(%)			*21.646%*
Final Model R^2(%)			***38.859%***
N = 716			
